# Prospective Observational Comparison of oXiris-Based Continuous Renal Replacement Therapy and Sustained Low-Efficiency Dialysis in Sepsis-Associated Acute Kidney Injury

**DOI:** 10.7759/cureus.118001

**Published:** 2026-10-16

**Authors:** Lingaraju K S, Shrisha Manjunath Hegde, Vindya B J, Prashanth C Dheerendra

**Affiliations:** 1 Department of Nephrology, Apollo Hospitals, Bengaluru, IND; 2 Department of Ophthalmology, Subbaiah Institute of Medical Sciences, Shivamogga, IND

**Keywords:** continuous renal replacement therapy, oxiris hemofilter, sepsis-associated acute kidney injury, septic shock, sustained low-efficiency dialysis

## Abstract

Background

Sepsis-associated acute kidney injury (AKI) frequently requires renal replacement therapy (RRT). Sustained low-efficiency dialysis (SLED) and oXiris-based continuous renal replacement therapy (CRRT) are established options, but prospective comparative data remain limited. This study compared ICU mortality and survival to hospital discharge without ongoing RRT between patients treated with oXiris-based CRRT and SLED.

Methods

This prospective observational study included 60 adults with septic shock-associated AKI requiring RRT in a tertiary ICU between March 2020 and March 2021; 30 received SLED and 30 received oXiris-based CRRT. Baseline characteristics, illness-severity scores, organ-support requirements, biochemical parameters, and clinical outcomes were assessed. Between-group comparisons were unadjusted; no multivariable or propensity-based adjustment was performed.

Results

Patients selected for oXiris-based CRRT had greater baseline hemodynamic and organ-support requirements, including lower mean arterial pressure (74.3 ± 11.9 vs 86.3 ± 9.6 mmHg; p < 0.001), more frequent use of three or more vasopressors (80.0% vs 20.0%; p < 0.001), and more frequent use of invasive mechanical ventilation (90.0% vs 63.3%; p = 0.015). Baseline Sequential Organ Failure Assessment (SOFA) scores were 11.0 ± 2.6 and 10.0 ± 1.7, respectively (p = 0.083), but this non-significant difference does not establish baseline equivalence. ICU mortality was higher with oXiris-based CRRT (83.3% vs 50.0%; RR 1.67, 95% CI 1.13-2.47; p = 0.006). Survival to discharge without ongoing RRT was lower with oXiris-based CRRT (10.0% vs 40.0%; RR 0.25, 95% CI 0.08-0.80; p = 0.007), but this endpoint was inherently survival-dependent. At 72 hours, SLED was associated with higher bicarbonate and lower lactate values; these comparisons used available observations and were not adjusted for baseline biochemical differences. ICU stay and RRT duration also differed, but interpretation was limited by high and unequal mortality.

Conclusion

SLED was associated with more favourable unadjusted outcomes, but these findings do not establish superiority. Non-randomised treatment allocation, marked baseline severity imbalance, survival-dependent outcome assessment, and differential mortality preclude causal attribution of the observed differences to RRT modality.

## Introduction

Sepsis is a major cause of critical illness, multiorgan dysfunction, and mortality, and acute kidney injury (AKI) is among its most serious complications [1]. Sepsis-associated AKI frequently coexists with hemodynamic instability, metabolic derangements, prolonged ICU stay, and increased organ-support requirements [1]. In patients with septic shock complicated by severe AKI, renal replacement therapy (RRT) may be required for refractory acidosis, electrolyte abnormalities, fluid overload, or uremic complications [2]. Continuous renal replacement therapy (CRRT) is commonly selected for hemodynamically unstable patients because it permits gradual fluid and solute removal [2].

Adsorptive hemofilters have been developed to extend the conventional renal-supportive role of CRRT by facilitating the removal of circulating endotoxins and inflammatory mediators [3]. The oXiris hemofilter is a modified AN69 membrane with diffusive and convective clearance properties combined with cytokine and endotoxin adsorption capabilities [3]. A systematic review and meta-analysis reported that oXiris-based CRRT may be associated with lower 28-day mortality and improvements in selected hemodynamic parameters in patients with sepsis. The certainty of these findings remains limited because the available evidence consisted predominantly of observational studies with small sample sizes and substantial clinical and methodological heterogeneity [3]. Separately, a cohort study comparing sustained low-efficiency dialysis (SLED) with CRRT in critically ill adults with AKI reported broadly comparable clinical outcomes, suggesting that SLED may be a feasible alternative in selected patients; however, its observational design did not permit definitive conclusions regarding comparative efficacy [4].

Comparative observational evidence suggests that oXiris-based CRRT may be associated with changes in vasopressor requirements, inflammatory markers, renal parameters, and short-term clinical outcomes in selected patients with sepsis [5]. Narrative evidence further describes its combined role in renal support and the adsorption of endotoxins and inflammatory mediators [6]. Clinical reports have demonstrated the feasibility of oXiris-based CRRT in critically ill patients with sepsis, AKI, and acute respiratory distress syndrome [7]. Single-centre experience has also shown improvements in selected hemodynamic and biochemical parameters during oXiris treatment, although mortality remained high among patients with severe septic shock [8].

Baseline illness severity, hemodynamic instability, and multiorgan dysfunction are important determinants of outcomes in patients receiving oXiris-based CRRT [9]. Experience from a resource-limited ICU has demonstrated the feasibility of oXiris hemoadsorption in sepsis-associated AKI, while also highlighting the limitations inherent in retrospective treatment comparisons [10]. Comparative studies of hemopurification filters have demonstrated differences in inflammatory and physiological responses without establishing clear superiority with respect to renal recovery or mortality [11]. Expert consensus therefore supports the selective use of oXiris in patients with sepsis or hyperinflammatory states, with consideration of treatment timing, clinical phenotype, and integration with standard sepsis management [12].

CRRT also presents pharmacological and operational challenges, including altered antimicrobial clearance and the need for individualized dosing during continuous venovenous hemodiafiltration [13]. SLED provides an alternative extended RRT strategy using slower rates of fluid and solute removal while retaining the practical advantages of conventional hemodialysis systems [14]. Available evidence indicates that SLED can correct acid-base, electrolyte, and volume abnormalities with acceptable hemodynamic tolerance in critically ill patients [14]. Long-term outcomes after CRRT are strongly influenced by illness severity and subsequent clinical trajectory, including survival, dialysis dependence, and renal recovery [15]. Multicentre observational data further show that oXiris is frequently used in patients with severe shock and multiple organ-support requirements, increasing the potential for confounding by indication in non-randomised comparisons [16]. Randomised crossover evidence has demonstrated that oXiris can reduce circulating endotoxin and cytokine concentrations, although biomarker removal alone does not establish a survival benefit [17].

Comparative evidence between SLED and CRRT in critically ill adults with AKI remains limited. Observational data indicate that SLED may be a feasible alternative to CRRT in selected patients but do not establish the superiority of either modality [4]. Evidence directly comparing oXiris-based CRRT with SLED in adults with sepsis-associated AKI is more limited. Broader evidence concerning RRT strategies in septic shock has not established consistent superiority of one modality across major clinical outcomes [14]. Studies of oXiris-based therapy have largely been observational, retrospective, or single-centre and have included heterogeneous critically ill populations [1,8,16]. Real-world use of oXiris also encompasses patients with varying indications, degrees of organ dysfunction, and requirements for renal and circulatory support, making treatment-selection effects particularly important when interpreting non-randomised outcomes [16]. Long-term outcomes among critically ill patients receiving CRRT are similarly influenced by underlying illness severity and subsequent clinical course [15,17].

The comparative effects of oXiris-based CRRT and SLED on ICU mortality, renal recovery, early hemodynamic response, duration of RRT, ICU length of stay, and resource utilisation therefore remain uncertain. Because treatment allocation was non-randomised, between-group differences were interpreted in the context of baseline illness severity and potential confounding by indication.

Objectives of the study

Among adults with septic shock-associated AKI requiring RRT in a tertiary ICU between March 2020 and March 2021, the primary objectives were to compare ICU mortality and renal recovery at hospital discharge between patients treated with oXiris-based CRRT and those treated with SLED. Secondary objectives were to compare vasopressor status and Sequential Organ Failure Assessment (SOFA) score at 72 hours; bicarbonate, lactate, and procalcitonin measurements at baseline and 72 hours; ICU length of stay; and duration of RRT. Given the non-randomised treatment allocation, these comparisons were exploratory and descriptive and were not intended to establish superiority or causal treatment effects.

## Materials and methods

Study design

This prospective observational study was conducted in the ICU of a tertiary care hospital. Clinical outcomes were compared between patients with septic shock-associated AKI treated with oXiris-based CRRT and those treated with SLED. Treatment allocation was non-randomised, and no prespecified random allocation procedure was used.

Study setting and duration

The study was conducted at Apollo Hospitals, Bannerghatta Road, Bengaluru, India, between March 2020 and March 2021.

Study population

Adult patients admitted to the ICU with septic shock-associated AKI requiring RRT were considered eligible. All consecutive patients who fulfilled the eligibility criteria and were treated with either oXiris-based CRRT or SLED during the study period were included. The treating physician selected the renal replacement modality according to the patient’s clinical condition, hemodynamic status, and treatment requirements. No prespecified allocation algorithm was used for modality assignment; therefore, selection of oXiris-based CRRT or SLED reflected the treating physician’s clinical judgement and was susceptible to confounding by indication. Modality assignment was not determined by the study investigators through randomisation or protocol-mandated allocation.

Eligibility criteria

Patients were included if they were older than 18 years, had septic shock-associated AKI, had a cardiovascular component score of 2 or more on the SOFA scale [18], required RRT using either oXiris-based CRRT or SLED, and had provided written informed consent either themselves or through a legally authorised representative.

Patients were enrolled with septic shock-associated AKI as defined within the original study framework, which referenced Sepsis-3 criteria for sepsis/septic shock and Kidney Disease: Improving Global Outcomes (KDIGO) criteria for AKI. The study additionally required a cardiovascular SOFA component score of ≥2 for inclusion. Although the original study framework described the Sepsis-3 septic shock and KDIGO AKI criteria, formal AKI staging and separate retrospective reclassification of each participant according to all components of these definitions were not performed for the present analysis.

Patients were excluded if they had chronic kidney disease, had received RRT within the preceding 30 days, were pregnant, had received immunosuppressive or systemic corticosteroid therapy within the preceding three months, had a recently diagnosed malignancy, were organ-transplant recipients, or had participated in another ongoing study within the preceding 30 days.

RRT protocol

CRRT was performed using continuous venovenous hemodiafiltration with the Prismaflex system (Gambro Lundia AB, Lund, Sweden) and oXiris hemofilter (Gambro Industries SAS, Meyzieu, France). A blood flow of 100-150 mL/min was maintained, with an intended effluent dose of 20-35 mL/kg/h. RRT was delivered through a temporary hemodialysis catheter, using either a 12-French triple-lumen catheter or an 11.5-French double-lumen catheter. The anatomical vascular-access site was not systematically recorded as a study variable.

Regional citrate or unfractionated heparin was used for anticoagulation. For CRRT, regional citrate anticoagulation was used with a target ionised calcium concentration of 0.9-1.1 mmol/L, while unfractionated heparin, when used, was administered as a continuous infusion of 500 IU/hour. No anticoagulation was used in patients with active bleeding or those considered to be at high risk of bleeding.

Patients in the SLED group underwent dialysis using a Fresenius 5008S hemodialysis machine with an FX5 dialyser (Fresenius Medical Care AG & Co. KGaA, Bad Homburg, Germany) at low efficiency. The blood flow rate was maintained at 100-150 mL/min, and the dialysate flow rate was 200-300 mL/min. Treatment duration was 6-8 hours per session. When anticoagulation was used, unfractionated heparin was administered as a 1,000-IU bolus followed by 500 IU until the end of the fifth treatment hour. In patients with active bleeding, saline dialysis was performed using 100-mL saline flushes every 30 minutes.

RRT initiation was determined by the treating clinical team according to the individual patient’s clinical condition and recognised indications for RRT in AKI. No fixed study-specific initiation threshold was applied, and exact intervals from ICU admission, septic shock onset, or AKI diagnosis to the initiation of RRT were not systematically evaluated as study variables. RRT discontinuation was determined by the treating team according to renal and overall clinical recovery rather than by a fixed study-specific cessation algorithm. The prescribed CRRT effluent target is reported above; actual delivered effluent dose, oXiris filter/circuit lifespan, total number of oXiris filters, total number and frequency of SLED sessions, and net ultrafiltration targets were not prospectively collected as study variables and therefore could not be reconstructed reliably.

Other aspects of sepsis management, including antimicrobial therapy, source control, fluid resuscitation, vasopressor therapy, and ventilatory support, were determined by the treating clinical team according to individual patient requirements and were not assigned by the study protocol. These co-interventions were not standardised according to RRT modality and were not analysed as protocol-defined between-group exposures.

Clinical and laboratory assessment

Baseline assessment consisted of age, sex, comorbidities, source of infection, and severity of illness. Illness severity was assessed using the SOFA score [18] and APACHE II score [19]. Both scores were assessed at recruitment, with APACHE II reflecting variables obtained within the first 24 hours of ICU admission; SOFA was reassessed at approximately 72 hours. Hemodynamic parameters, vasopressor use, and ventilatory support were also documented during this period.

Laboratory parameters comprised CBC, renal function tests, serum electrolytes, arterial blood gas analysis, serum bicarbonate, serum lactate, procalcitonin, and routine urine investigations. These parameters were evaluated at baseline and 72 hours. Blood and urine cultures were obtained as needed. Laboratory investigations were performed as part of routine hospital clinical care. Routine biochemical investigations were analysed using an AU480 chemistry analyser (Beckman Coulter, Inc., Brea, California, USA), and CBCs were analysed using a DxH 600 hematology analyser (Beckman Coulter, Inc., Brea, California, USA) in the institution’s National Accreditation Board for Testing and Calibration Laboratories (NABL)-accredited laboratory; both instruments were calibrated according to institutional protocol. The specific analytical platform and assay characteristics used for procalcitonin were not recorded as prespecified study variables.

Treatment allocation could not be blinded, and no separate blinded outcome-adjudication procedure was used. Clinical and laboratory information was collected prospectively during the study period; however, duplicate abstraction, inter-rater reliability assessment, and formal blinded adjudication were not components of the study protocol.

Outcomes and follow-up

The primary outcomes were ICU mortality, defined as all-cause death occurring during the index ICU admission, and renal recovery at hospital discharge, operationally defined as becoming dialysis-independent at the time of hospital discharge. Because dialysis independence at discharge could occur only among patients surviving to discharge, this outcome was inherently survival-dependent and was not interpreted as kidney recovery conditional on survival.

Secondary outcomes included vasopressor status and SOFA score at 72 hours; biochemical measurements of bicarbonate, lactate, and procalcitonin at baseline and 72 hours; duration of RRT; and ICU length of stay. Requirement for mechanical ventilation was treated as a baseline organ-support characteristic in the current comparative analysis.

Surviving patients were evaluated at one and three months after hospital discharge for renal function, dialysis dependence, readmission, and mortality. Given the small number of survivors, particularly in the oXiris-based CRRT group, these follow-up findings were treated as descriptive and were not used for formal comparative inference.

For 72-hour assessments, 25 patients in the SLED group and 18 patients in the oXiris-based CRRT group remained alive and evaluable. Five SLED patients and 12 oXiris-based CRRT patients died before the 72-hour assessment. Patients who died before the scheduled assessment contributed no post-death measurements, and no values were imputed after death.

ICU length of stay was evaluated among survivors and was therefore treated as a survivor-restricted descriptive outcome. RRT duration was reported as an observed treatment-duration measure and was compared between groups using the original unadjusted analysis. No competing-risk analysis was performed. Both duration outcomes were interpreted cautiously because of the substantial and unequal mortality between treatment groups.

Ethical considerations

The study protocol was reviewed and approved by the Institutional Ethics Committee of Apollo Hospitals, Bengaluru, at its meeting held on March 2, 2020 (approval/reference number: AHB/SRC/IEC/DNB/006/2020; formal approval letter dated June 3, 2020). The study was conducted in accordance with the principles of the Declaration of Helsinki and applicable institutional ethical standards. Written informed consent was obtained from all participants or their legally authorised representatives before enrollment.

Statistical analysis

SPSS Statistics for Windows, version 17.0 (SPSS Inc., Chicago, Illinois, USA) was used for statistical analysis. Continuous variables were expressed as mean ± SD or median and range, as appropriate, whereas categorical variables were presented as frequency (n) and percentage (%). Between-group comparisons of continuous variables were performed using the independent-samples t-test. Categorical variables were compared using the Pearson chi-square test, while Fisher’s exact test was used when expected cell counts were small. For inferential comparisons, the corresponding test statistic (t or χ², as applicable), statistical test, and two-sided p-value were reported. For Fisher’s exact test, ORs were reported where calculable; when a zero cell precluded direct estimation, the OR was reported as not estimable.

For the principal binary outcomes of ICU mortality and renal recovery at hospital discharge, relative risks with 95% CIs were calculated using the SLED group as the reference group. Survivor-conditional dialysis independence was additionally reported descriptively to clarify the influence of differential mortality on the renal-recovery endpoint.

For outcomes assessed at 72 hours, an available-case approach was used. The evaluable population comprised patients surviving to the 72-hour assessment; 5/30 SLED patients and 12/30 oXiris-based CRRT patients died before this assessment. No post-death values were imputed, and the corresponding denominators were reported for each 72-hour comparison.

ICU length of stay was treated as a survivor-restricted descriptive outcome in the revised analysis. No revised inferential comparison was performed for ICU length of stay because the original outcome was reported among survivors rather than the complete treatment groups. No competing-risk analysis was performed for duration of RRT.

No multivariable regression or propensity-score method was performed; therefore, all between-group comparisons were unadjusted. Given the limited sample size and substantial baseline differences in hemodynamic and organ-support requirements between treatment groups, adjusted modelling was considered unlikely to provide sufficiently robust estimates and carried a substantial risk of model instability and overfitting. The analyses were therefore considered exploratory and hypothesis-generating rather than estimates of causal treatment effects.

No formal a priori sample-size calculation was performed, and no adjustment for multiplicity was applied. Formal normality-testing results were not available from the original analysis. Therefore, reported parametric comparisons, particularly for duration and biomarker variables that may have been skewed, were retained as originally analysed and interpreted cautiously rather than being retrospectively reclassified using unavailable patient-level distributional data.

## Results

Study population and baseline characteristics

There were 60 patients with sepsis-associated AKI, with 30 receiving SLED and 30 receiving oXiris-based CRRT. The groups were broadly similar in age, sex, and the major recorded comorbidities. Diabetes mellitus was more common in the oXiris-based CRRT group, with 22/30 (73.3%) patients versus 16/30 (53.3%) in the SLED group, but the difference was not statistically significant (Pearson chi-square test, χ² = 2.58, p = 0.108). Hypertension was present in 15/30 (50.0%) patients in each group, ischemic heart disease in 17/30 (56.7%) versus 14/30 (46.7%), and chronic obstructive pulmonary disease in 6/30 (20.0%) versus 5/30 (16.7%) in the oXiris-based CRRT and SLED groups, respectively (Table 1). Although demographic characteristics and the major recorded comorbidities were broadly comparable, subsequent outcome comparisons should be interpreted in the context of clinically important baseline differences in hemodynamic instability and organ-support requirements.

**Table 1:** Baseline demographic and clinical characteristics of the study population. CRRT: Continuous renal replacement therapy; SLED: Sustained low-efficiency dialysis. Continuous variables were compared using the independent-samples t-test, and categorical variables were compared using the Pearson chi-square test. Test statistics are reported as t or χ², as applicable. A two-sided p-value < 0.05 was considered statistically significant. This table describes demographic characteristics and comorbidities; clinically important baseline differences in hemodynamic instability and organ-support requirements are presented separately and should be considered when interpreting subsequent outcome comparisons.

Characteristic	Category	SLED (n = 30)	CRRT (n = 30)	Test statistic	Statistical test	p-value
Age, years	Mean ± SD	61.0 ± 10.5	63.1 ± 11.6	t = -0.74	Independent-samples t-test	0.465
Sex, n (%)	Female	11 (36.7%)	8 (26.7%)	χ² = 0.69	Pearson chi-square test	0.405
	Male	19 (63.3%)	22 (73.3%)	-	-	-
Diabetes mellitus, n (%)	Present	16 (53.3%)	22 (73.3%)	χ² = 2.58	Pearson chi-square test	0.108
	Absent	14 (46.7%)	8 (26.7%)	-	-	-
Hypertension, n (%)	Present	15 (50.0%)	15 (50.0%)	χ² = 0.00	Pearson chi-square test	1
	Absent	15 (50.0%)	15 (50.0%)	-	-	-
Ischemic heart disease, n (%)	Present	14 (46.7%)	17 (56.7%)	χ² = 0.60	Pearson chi-square test	0.438
	Absent	16 (53.3%)	13 (43.3%)	-	-	-
Chronic liver disease, n (%)	Present	10 (33.3%)	7 (23.3%)	χ² = 0.74	Pearson chi-square test	0.39
	Absent	20 (66.7%)	23 (76.7%)	-	-	-
Chronic obstructive pulmonary disease, n (%)	Present	5 (16.7%)	6 (20.0%)	χ² = 0.11	Pearson chi-square test	0.739
	Absent	25 (83.3%)	24 (80.0%)	-	-	-

Illness severity and hemodynamic status at baseline

Patients receiving oXiris-based CRRT had greater hemodynamic instability at baseline. Mean arterial pressure was significantly lower in the oXiris-based CRRT group than in the SLED group (74.3 ± 11.9 versus 86.3 ± 9.6 mmHg; independent-samples t-test, t = 4.30, p < 0.001). The requirement for three or more vasopressors was also substantially higher in the oXiris-based CRRT group (24/30 (80.0%) versus 6/30 (20.0%); Pearson chi-square test, χ² = 21.60, p < 0.001).

APACHE II scores were similar between the groups. The mean baseline SOFA score was numerically higher in the oXiris-based CRRT group (11.0 ± 2.6 versus 10.0 ± 1.7), but the difference was not statistically significant (independent-samples t-test, t = -1.76, p = 0.083) (Table 2). These marked baseline differences in hemodynamic instability and vasopressor requirements indicate that patients selected for oXiris-based CRRT were clinically more unstable at baseline, which should be considered when interpreting subsequent unadjusted differences in mortality, renal recovery, and other clinical outcomes. These differences in hemodynamic and illness-severity parameters are illustrated in Figure 1.

**Table 2:** Illness severity and hemodynamic status at baseline. APACHE II: Acute Physiology and Chronic Health Evaluation II; CRRT: Continuous renal replacement therapy; SLED: Sustained low-efficiency dialysis; SOFA: Sequential Organ Failure Assessment. Continuous variables were compared using the independent-samples t-test, and categorical variables were compared using the Pearson chi-square test. Test statistics are reported as t or χ², as applicable. A two-sided p-value < 0.05 was considered statistically significant. The substantially lower mean arterial pressure and markedly greater vasopressor requirement in the oXiris-based CRRT group indicate an important baseline hemodynamic imbalance and should be considered when interpreting subsequent unadjusted clinical outcome comparisons.

Parameter	SLED (n = 30)	CRRT (n = 30)	Test statistic	Statistical test	p-value
Mean arterial pressure, mmHg, mean ± SD	86.3 ± 9.6	74.3 ± 11.9	t = 4.30	Independent-samples t-test	< 0.001
APACHE II score, mean ± SD	23.5 ± 2.7	23.7 ± 4.0	t = -0.23	Independent-samples t-test	0.821
Baseline SOFA score, mean ± SD	10.0 ± 1.7	11.0 ± 2.6	t = -1.76	Independent-samples t-test	0.083
Requirement for ≥3 vasopressors at baseline, n (%)	6 (20.0%)	24 (80.0%)	χ² = 21.60	Pearson chi-square test	< 0.001

**Figure 1:**
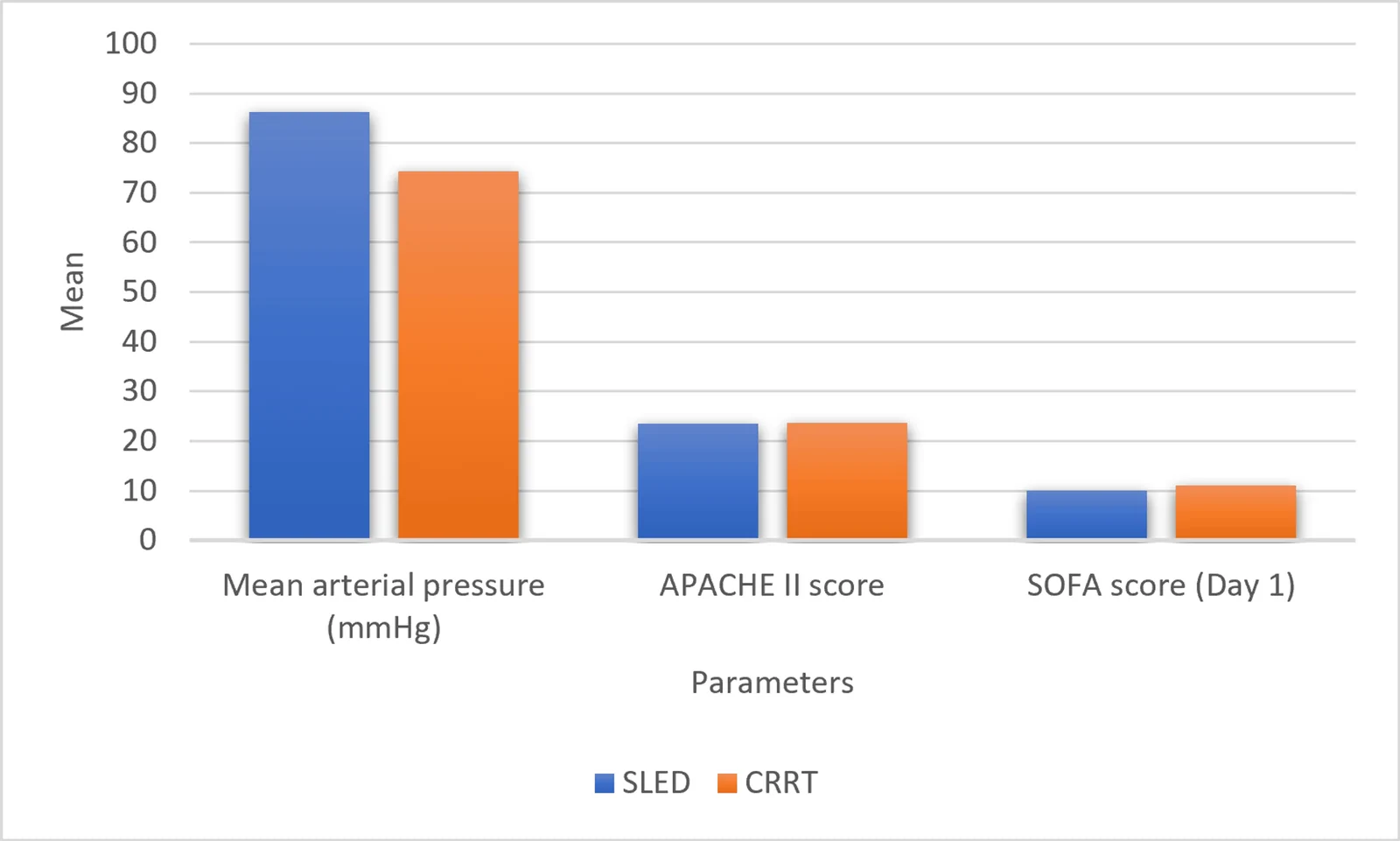
Comparison of hemodynamic and illness-severity parameters at baseline. APACHE II: Acute Physiology and Chronic Health Evaluation II; CRRT: Continuous renal replacement therapy; SLED: Sustained low-efficiency dialysis; SOFA: Sequential Organ Failure Assessment. The figure shows the mean values of mean arterial pressure, APACHE II score, and baseline SOFA score in the SLED (n = 30) and oXiris-based CRRT (n = 30) groups.

Clinical course during the first 72 hours

At 72 hours, the proportion of evaluable patients who were free from vasopressor support was higher in the SLED group than in the oXiris-based CRRT group, 12/25 (48.0%) versus 4/18 (22.2%); however, the difference was not statistically significant (Pearson chi-square test, χ² = 2.98, p = 0.084). The mean day 3 SOFA score was significantly lower among patients receiving SLED (6.1 ± 3.5 versus 11.2 ± 3.1; independent-samples t-test, t = -4.94, p < 0.001).

Invasive mechanical ventilation at baseline was required in 19/30 (63.3%) patients in the SLED group and 27/30 (90.0%) in the oXiris-based CRRT group (Pearson chi-square test, χ² = 5.96, p = 0.015). These findings were consistent with the greater baseline respiratory and hemodynamic support requirements in patients selected for oXiris-based CRRT. Accordingly, the observed 72-hour differences should be interpreted as unadjusted associations in the context of substantial baseline differences in illness severity and organ-support requirements rather than as evidence of a treatment effect (Table 3).

**Table 3:** Clinical course during the first 72 hours. CRRT: Continuous renal replacement therapy; SLED: Sustained low-efficiency dialysis; SOFA: Sequential Organ Failure Assessment. For the 72-hour assessments, 25 patients in the SLED group and 18 patients in the oXiris-based CRRT group were alive and evaluable; five SLED patients and 12 oXiris-based CRRT patients died before the 72-hour assessment. No post-death values were imputed. The invasive mechanical ventilation comparison reflects status at baseline and therefore uses the full baseline population (n = 30 per group). All comparisons were unadjusted and should be interpreted in the context of baseline differences in clinical severity and differential survival to 72 hours.

Parameter	SLED	oXiris-based CRRT	Test statistic	Statistical test	p-value
Vasopressor-free at 72 hours, n/N (%)	12/25 (48.0%)	4/18 (22.2%)	χ² = 2.98	Pearson chi-square test	0.084
SOFA score at 72 hours, mean ± SD	6.1 ± 3.5 (n = 25)	11.2 ± 3.1 (n = 18)	t = -4.94	Independent-samples t-test	< 0.001
Invasive mechanical ventilation at baseline, n/N (%)	19/30 (63.3%)	27/30 (90.0%)	χ² = 5.96	Pearson chi-square test	0.015

Biochemical response

Both groups had marked metabolic abnormalities at baseline. Mean serum bicarbonate was lower in the oXiris-based CRRT group, although the between-group difference was not statistically significant (12.4 ± 3.2 versus 14.3 ± 4.2 mEq/L; independent-samples t-test, t = 1.97, p = 0.054). At 72 hours, bicarbonate was significantly higher in the SLED group (20.3 ± 4.2 versus 13.8 ± 7.8 mEq/L; independent-samples t-test, t = 3.53, p = 0.001). Because the 72-hour comparison was not adjusted for baseline bicarbonate values, it should not be interpreted as evidence of a differential treatment response.

Baseline serum lactate was significantly higher in the oXiris-based CRRT group (7.6 ± 3.7 versus 5.1 ± 3.1 mmol/L; independent-samples t-test, t = -2.84, p = 0.006). At 72 hours, lactate remained higher in the oXiris-based CRRT group than in the SLED group (3.8 ± 3.3 versus 2.0 ± 1.5 mmol/L; independent-samples t-test, t = -2.41, p = 0.020). Because baseline lactate already differed significantly between the groups and no baseline-adjusted analysis was performed, the 72-hour difference cannot be interpreted as evidence of a greater lactate response with either modality. The greater baseline hemodynamic instability of patients receiving oXiris-based CRRT further limits the causal interpretation of this comparison.

At 72 hours, the mean procalcitonin concentration was 24.1 ± 13.8 ng/mL in the SLED group and 19.4 ± 7.7 ng/mL in the oXiris-based CRRT group, with no statistically significant between-group difference (independent-samples t-test, t = 1.63, p = 0.109) (Table 4). As with the other 72-hour biochemical measures, this comparison was unadjusted and should be interpreted as a cross-sectional between-group comparison at that time point rather than as evidence of differential biochemical improvement.

**Table 4:** Biochemical parameters at baseline and 72 hours. CRRT: Continuous renal replacement therapy; SLED: Sustained low-efficiency dialysis. Between-group comparisons at each time point were performed using the independent-samples t-test. Baseline analyses included all 30 patients in each group. At 72 hours, analyses were restricted to patients who remained alive and evaluable (SLED, n = 25; oXiris-based CRRT, n = 18); five SLED patients and 12 oXiris-based CRRT patients died before the 72-hour assessment, and no post-death values were imputed. The 72-hour comparisons were unadjusted for baseline biochemical values and should therefore be interpreted as survivor-conditioned, cross-sectional between-group comparisons rather than as evidence of a differential treatment response. A two-sided p-value < 0.05 was considered statistically significant.

Parameter	Time point	SLED	oXiris-based CRRT	Test statistic	Statistical test	p-value
Bicarbonate, mEq/L, mean ± SD	Baseline	14.3 ± 4.2 (n=30)	12.4 ± 3.2 (n=30)	t = 1.97	Independent-samples t-test	0.054
	72 hours	20.3 ± 4.2 (n=25)	13.8 ± 7.8 (n=18)	t = 3.53	Independent-samples t-test	0.001
Lactate, mmol/L, mean ± SD	Baseline	5.1 ± 3.1 (n=30)	7.6 ± 3.7 (n=30)	t = -2.84	Independent-samples t-test	0.006
	72 hours	2.0 ± 1.5 (n=25)	3.8 ± 3.3 (n=18)	t = -2.41	Independent-samples t-test	0.020
Procalcitonin, ng/mL, mean ± SD	Baseline	35.9 ± 26.5 (n=30)	38.5 ± 20.0 (n=30)	t = -0.43	Independent-samples t-test	0.67
	72 hours	24.1 ± 13.8 (n=25)	19.4 ± 7.7 (n=18)	t = 1.30	Independent-samples t-test	0.200

Serum bicarbonate increased from baseline to 72 hours in both groups and was significantly higher in the SLED group at 72 hours (independent-samples t-test, t = 3.53, p = 0.001). Lactate concentrations decreased in both groups and remained significantly lower in the SLED group at 72 hours (t = -2.41, p = 0.020). Procalcitonin concentrations also decreased over 72 hours, although the between-group difference at 72 hours was not statistically significant (t = 1.30, p = 0.200). These biochemical comparisons were unadjusted and should be interpreted in the context of the higher baseline lactate concentration and greater hemodynamic instability in the oXiris-based CRRT group. These biochemical changes during the first 72 hours are illustrated in Figure 2.

**Figure 2:**
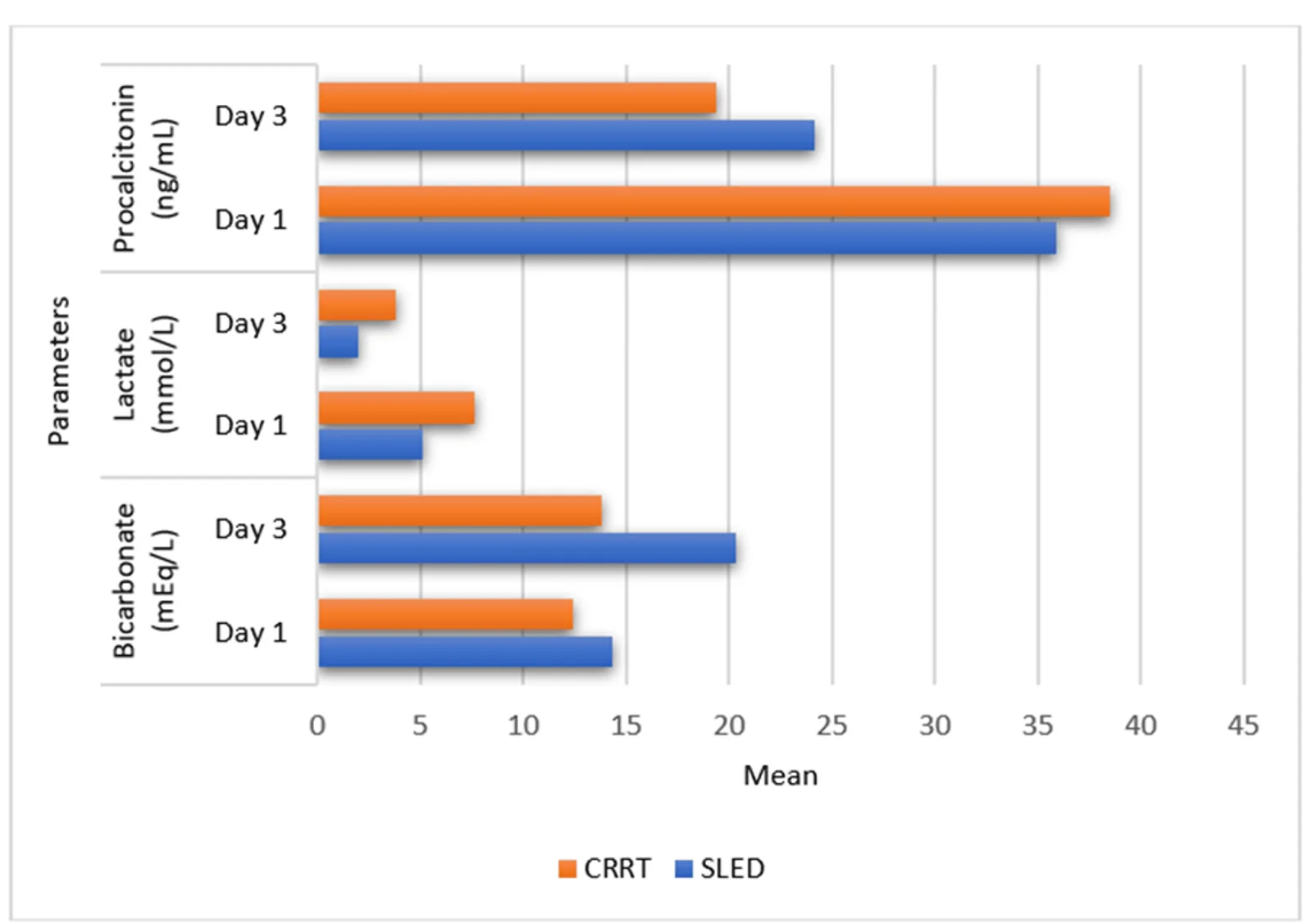
Changes in biochemical parameters over 72 hours. CRRT: Continuous renal replacement therapy; SLED: Sustained low-efficiency dialysis. The figure presents the mean serum bicarbonate, lactate, and procalcitonin concentrations in the SLED and oXiris-based CRRT groups at baseline and 72 hours.

ICU outcomes and resource utilisation

ICU mortality was significantly higher in the oXiris-based CRRT group than in the SLED group (25/30 (83.3%) versus 15/30 (50.0%); RR for oXiris-based CRRT versus SLED, 1.67; 95% CI, 1.13-2.47; Pearson chi-square test, χ² = 7.50, p = 0.006). Renal recovery, defined as dialysis independence at hospital discharge, occurred in 12/30 (40.0%) patients in the SLED group and 3/30 (10.0%) patients in the oXiris-based CRRT group (RR for oXiris-based CRRT versus SLED, 0.25; 95% CI, 0.08-0.80; Pearson chi-square test, χ² = 7.20, p = 0.007). Because this endpoint was dependent on survival to discharge, survivor-conditional proportions were also examined descriptively: 12/15 (80.0%) SLED survivors and 3/5 (60.0%) oXiris-based CRRT survivors were dialysis-independent at discharge. These survivor-conditional proportions were not subjected to a separate inferential comparison. The primary effect estimates were unadjusted and should be interpreted in the context of substantial baseline imbalance and differential mortality between the treatment groups.

Among survivors, mean ICU length of stay was 23.6 ± 8.3 days in the oXiris-based CRRT group and 4.9 ± 5.5 days in the SLED group. Because ICU length of stay was assessed only among survivors, this outcome is presented descriptively, and no revised inferential comparison was performed. The mean duration of RRT was greater among patients receiving oXiris-based CRRT than among those receiving SLED (69.1 ± 30.1 versus 20.8 ± 6.9 hours; independent-samples t-test, t = -8.57, p < 0.001) (Table 5). The duration outcomes should be interpreted cautiously because of differential survival and substantial baseline clinical instability, and neither difference should be interpreted as a causal effect of RRT modality.

**Table 5:** Major clinical outcomes and resource utilisation. CRRT: Continuous renal replacement therapy; RRT: Renal replacement therapy; SLED: Sustained low-efficiency dialysis. Relative risks with 95% CIs are reported for ICU mortality and dialysis independence at hospital discharge, comparing the oXiris-based CRRT group with the SLED group. All inferential comparisons were unadjusted. ICU length of stay was reported among survivors only (SLED, n=15; oXiris-based CRRT, n=5) and is therefore presented descriptively. Dialysis independence at hospital discharge was a survival-dependent outcome.

Outcome	SLED	oXiris-based CRRT	Effect estimate (95% CI)	Test statistic	Statistical test	p-value
ICU mortality, n/N (%)	15/30 (50.0%)	25/30 (83.3%)	RR 1.67 (95% CI 1.13-2.47)	χ² = 7.50	Pearson chi-square test	0.006
Dialysis independence at hospital discharge, n/N (%)	12/30 (40.0%)	3/30 (10.0%)	RR 0.25 (95% CI 0.08-0.80)	χ² = 7.20	Pearson chi-square test	0.007
ICU length of stay among survivors, days, mean ± SD	4.9 ± 5.5 (n=15)	23.6 ± 8.3 (n=5)	-	-	Descriptive comparison	-
Duration of RRT, hours, mean ± SD	20.8 ± 6.9 (n=30)	69.1 ± 30.1 (n=30)	-	t = -8.57	Independent-samples t-test	<0.001

Microbiological profile

Gram-negative bacilli were the predominant organisms in both groups, accounting for 21/30 (70.0%) of cases in the SLED group and 27/30 (90.0%) in the oXiris-based CRRT group. Gram-positive cocci accounted for 6/30 (20.0%) and 2/30 (6.7%) of cases, respectively. Candida species, tuberculosis, and no organism isolated each accounted for 1/30 (3.3%) of cases in the SLED group, while tuberculosis accounted for 1/30 (3.3%) of cases in the oXiris-based CRRT group. The microbiological distribution in both treatment groups is illustrated in Figure 3.

**Figure 3:**
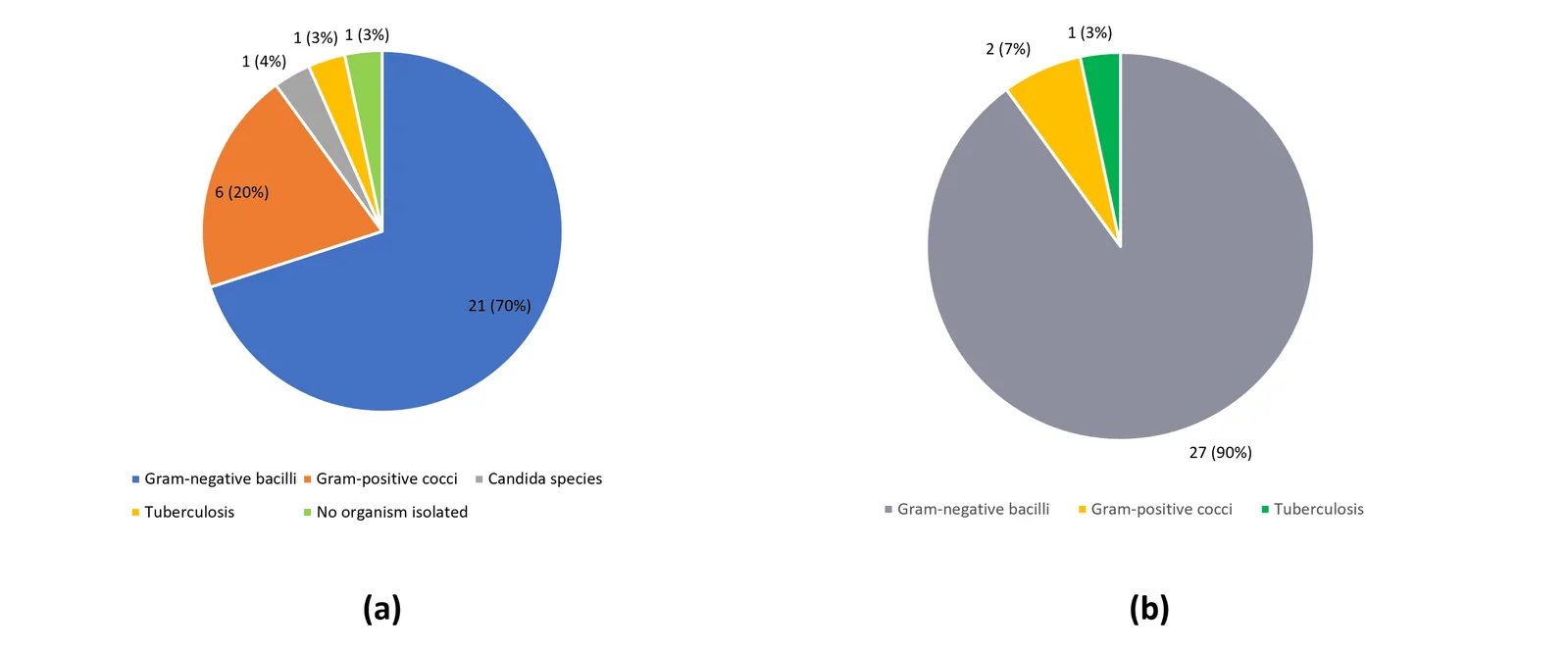
Microbiological profile of the study groups: (a) SLED and (b) oXiris-based CRRT. Values are presented as n (%). CRRT: Continuous renal replacement therapy; n: Number of patients; SLED: Sustained low-efficiency dialysis.

Source of infection

Community-acquired pneumonia was the most frequently recorded source of infection, occurring in 8/30 (26.7%) patients receiving SLED and 12/30 (40.0%) patients receiving oXiris-based CRRT. Urinary tract infection was identified in 6/30 (20.0%) and 5/30 (16.7%) patients, respectively. Spontaneous bacterial peritonitis occurred in 3/30 (10.0%) patients in each group.

Other sources included ventilator-associated pneumonia, lower-limb cellulitis, perianal abscess, abdominal sepsis or intestinal obstruction, and disseminated tuberculosis. None of the documented infection-source categories differed significantly between the SLED and oXiris-based CRRT groups (two-sided Fisher’s exact test; all p > 0.05) (Table 6).

**Table 6:** Sources of infection in the study population. CRRT: Continuous renal replacement therapy; SLED: Sustained low-efficiency dialysis. Categorical comparisons were performed using the two-sided Fisher’s exact test because of small expected cell counts. ORs are provided as effect estimates where calculable. A two-sided p-value <0.05 was considered statistically significant. None of the infection-source distributions differed significantly between the two groups. The source of infection was not documented in three patients in the SLED group and four patients in the oXiris-based CRRT group. No inferential comparison was performed for this missing-data category.

Source of infection	SLED (n = 30), n (%)	oXiris-based CRRT (n = 30), n (%)	OR	Statistical test	p-value
Community-acquired pneumonia	8 (26.7%)	12 (40.0%)	0.55	Fisher’s exact test	0.412
Ventilator-associated pneumonia	2 (6.7%)	2 (6.7%)	1	Fisher’s exact test	1
Urinary tract infection	6 (20.0%)	5 (16.7%)	1.25	Fisher’s exact test	1
Spontaneous bacterial peritonitis	3 (10.0%)	3 (10.0%)	1	Fisher’s exact test	1
Lower-limb cellulitis	4 (13.3%)	1 (3.3%)	4.46	Fisher’s exact test	0.353
Perianal abscess	2 (6.7%)	1 (3.3%)	2.07	Fisher’s exact test	1
Abdominal sepsis or intestinal obstruction	1 (3.3%)	2 (6.7%)	0.48	Fisher’s exact test	1
Disseminated tuberculosis	1 (3.3%)	0 (0.0%)	Not estimable	Fisher’s exact test	1
Source of infection not documented	3 (10.0%)	4 (13.3%)	-	-	-

## Discussion

This is a prospective observational study comparing oXiris-based CRRT with SLED in patients with sepsis-associated AKI. Patients receiving oXiris-based CRRT showed higher ICU mortality, a lower proportion surviving to hospital discharge without an ongoing requirement for RRT, a longer duration of RRT, and a longer ICU stay. The renal endpoint was inherently dependent on survival and should therefore not be interpreted as renal recovery conditional on survival. These unadjusted differences should be interpreted cautiously because patients selected for oXiris-based CRRT had substantially greater baseline hemodynamic and organ-support requirements, including lower mean arterial pressure, markedly greater vasopressor use, and more frequent invasive mechanical ventilation. The mean baseline SOFA score was higher in the oXiris-based CRRT group (11.0 ± 2.6 versus 10.0 ± 1.7), although the difference did not reach statistical significance; given the limited sample size, this finding should not be interpreted as evidence of equivalence or comparability in illness severity. Long-term outcomes of critically ill patients receiving CRRT also indicate that the probability of survival, dialysis independence, and renal recovery is highly dependent on underlying illness severity and subsequent clinical course [15].

The marked baseline imbalance between treatment groups indicates substantial potential for confounding by indication. In clinical practice, oXiris-based CRRT is frequently selected for patients with profound hemodynamic instability, severe organ dysfunction, and greater requirements for continuous organ support. Real-world observational data similarly indicate that patients receiving oXiris often represent a more severely ill population with complex multiorgan dysfunction [16]. Consequently, the higher mortality and lower proportion surviving to discharge without ongoing RRT observed in the oXiris-based CRRT group should not be interpreted as evidence that oXiris-based CRRT or the oXiris membrane itself produces inferior clinical outcomes. APACHE II scores were nearly identical between groups despite marked differences in mean arterial pressure, vasopressor requirements, and invasive mechanical ventilation. This divergence suggests that global severity scores alone may not have fully captured the hemodynamic and organ-support imbalance influencing modality selection, further supporting the possibility of residual confounding despite apparently similar aggregate severity scores.

The timing of RRT initiation and the timing of oXiris initiation may also have influenced the observed outcomes. Differences in the interval between clinical deterioration or indication for RRT and initiation of SLED or oXiris-based CRRT could have affected the clinical course, particularly because oXiris-based CRRT may have been initiated in patients with more advanced hemodynamic instability or organ dysfunction. Treatment timing was not systematically evaluated as a determinant of outcome in the present study; therefore, its potential contribution to the observed between-group differences cannot be excluded and represents an additional source of residual confounding.

The biological rationale for oXiris remains clinically relevant despite uncertainty regarding patient-centred outcomes. Randomised crossover evidence has demonstrated that the oXiris membrane can reduce circulating endotoxin and inflammatory cytokine concentrations in patients with septic shock [17]. More recent randomised evidence has likewise shown biological effects of enhanced adsorption haemofiltration in severe refractory septic shock, although a clear mortality benefit has not been established [20]. Clinical reports have also demonstrated the use of CRRT as part of multimodal management for septic shock with AKI, respiratory failure, and severe metabolic disturbances [21]. In other hyperinflammatory critical illnesses, pilot data indicate that adsorptive hemofiltration may lower levels of selected inflammatory mediators, although changes in biomarkers cannot be assumed to translate into improvements in survival or renal recovery [22].

At 72 hours, bicarbonate was higher and lactate was lower in the SLED group; however, these were unadjusted between-group comparisons rather than evidence of differential biochemical improvement. Baseline lactate was already significantly higher in the oXiris-based CRRT group, and the 72-hour lactate comparison was not adjusted for this baseline difference. In addition, 72-hour analyses were conditional on patients having evaluable observations at that time point, with different evaluable denominators between groups. These findings are therefore susceptible to baseline confounding, differential attrition, and survivorship bias and should not be interpreted as intrinsic physiological advantages of SLED. During oXiris-based CRRT, Theisen BE et al. reported simultaneous removal of endotoxins, inflammatory mediators, and uremic toxins, although mortality remained substantial among patients with advanced septic shock [23]. Zhai Y et al. also reported improvements in inflammatory and clinical parameters after oXiris treatment without establishing a clear survival advantage [24]. Taken together, these observations support the use of extracorporeal mediator removal as an adjunct to, rather than a replacement for, source control, appropriate antimicrobial therapy, and hemodynamic stabilisation.

Registry data also indicate that patients receiving CRRT are frequently more severely ill than those managed with intermittent or hybrid renal replacement strategies, complicating direct comparisons of mortality and renal outcomes across modalities [25]. A case report has also described combined extracorporeal carbon dioxide removal, RRT, and blood purification in a patient with severe inhalation injury, although such evidence is not directly comparable with adult septic AKI cohorts [26]. Hemoadsorption research in the neonatal and paediatric population further suggests that it is technically feasible, but these findings cannot be directly extrapolated to adults with sepsis-associated AKI because of substantial differences in physiology, disease patterns, and treatment indications [27]. Randomised evidence evaluating enhanced adsorption approaches has similarly demonstrated measurable biological effects without establishing a consistent mortality benefit. In the ENDoX bicentric randomised controlled trial, enhanced adsorption haemofiltration produced biological effects in patients with severe refractory septic shock but did not demonstrate a clear mortality advantage [20].

The observed differences in ICU length of stay and RRT duration should likewise be interpreted cautiously. Death was not modelled as a competing event, and the marked difference in mortality between groups could influence the time available to accrue these duration outcomes. Consequently, the longer ICU stay and RRT duration observed in the oXiris-based CRRT group are descriptive associations and cannot be interpreted as adverse effects of the modality itself.

The findings of the present study support an individualised approach to RRT modality selection rather than the superiority of one strategy. Modality choice should consider hemodynamic stability, degree of organ dysfunction, therapeutic objectives, anticipated need for continuous fluid and solute control, resource availability, and institutional expertise. SLED may be a feasible option for selected patients who can tolerate prolonged intermittent therapy, whereas oXiris-based CRRT may be considered when continuous renal support and adsorptive therapy are clinically indicated. The present observational data cannot determine whether either modality independently improves survival or kidney recovery among survivors.

Strengths, limitations, and future directions

This study has several strengths. Its prospective observational design permitted contemporaneous collection of clinical and laboratory data and provided a direct head-to-head comparison of oXiris-based CRRT and SLED in a clinically important population for which prospective comparative evidence remains limited. Both treatment modalities were described using quantified treatment parameters, including device type, blood-flow rates, dialysate or effluent prescriptions, treatment duration, and anticoagulation strategy. Statistical reporting was transparent, with the statistical test, test statistic, and p-value reported for inferential comparisons, and the interpretation consistently avoided causal or superiority claims in view of the non-randomised treatment allocation.

This study has a few important limitations. It was conducted at a single centre and used a non-randomised observational design, thereby increasing the risk of selection bias, confounding by indication, and limited external validity. In addition, septic shock and AKI were identified according to the treating team’s clinical assessment and study eligibility criteria rather than prospectively documented formal Sepsis-3 and KDIGO classifications, which may limit the reproducibility of the case definitions and comparison with other cohorts. The limited number of patients decreases the power of the analysis and limits the reliability of subgroup analyses. The renal outcome was defined as survival to hospital discharge without an ongoing requirement for RRT and was therefore dependent on survival; although survivor-conditional dialysis independence was additionally reported descriptively, the small number of survivors, particularly in the oXiris-based CRRT group, precluded robust inferential comparison. Accordingly, the full-cohort difference should not be interpreted as an isolated effect on renal recovery.

Residual confounding remains a major limitation when interpreting mortality, survival without ongoing RRT, ICU length of stay, and RRT-duration differences. The timing of RRT initiation and oXiris initiation was not systematically evaluated as a determinant of outcome. Differences in treatment timing, particularly initiation in the setting of more advanced shock or organ dysfunction, may therefore have contributed to the observed between-group differences. Co-interventions, including antimicrobial treatment, source-control procedures, fluid administration, vasopressor management, and ventilatory support, were determined clinically rather than standardised by study group and may have introduced additional residual confounding.

Deaths were not modelled as competing events for ICU length of stay or duration of RRT. Given the high and unequal mortality between groups, death could truncate these duration outcomes and complicate interpretation of their observed differences. The longer ICU stay in the oXiris-based CRRT group despite its higher mortality should therefore be interpreted as an unadjusted descriptive finding rather than evidence of a modality-specific effect.

The 72-hour analyses used available-case denominators, with fewer evaluable patients than at baseline, particularly in the oXiris-based CRRT group. Because unavailable observations may have been associated with clinical deterioration or other non-random processes, differential attrition may have introduced additional bias. These analyses were also conditional on remaining evaluable at 72 hours and may therefore be susceptible to survivorship bias. Baseline lactate differed significantly between groups, and the 72-hour biochemical comparisons were not adjusted for baseline values, limiting interpretation of these measurements as treatment responses. Formal normality-testing results were not available, and some duration and biomarker variables may have been skewed; parametric comparisons for these outcomes should therefore be interpreted cautiously. No formal a priori sample-size calculation or adjustment for multiple comparisons was performed, increasing the possibility of imprecision and chance findings across the exploratory analyses.

No measurements of endotoxin or cytokines were made directly. Although surviving patients underwent one- and three-month follow-up, the small number of survivors, particularly in the oXiris-based CRRT group, limited meaningful comparative inference regarding longer-term dialysis dependence and kidney recovery. The enrolment period overlapped with the COVID-19 pandemic, and SARS-CoV-2 status was not analysed as a prespecified study variable; possible pandemic-related case-mix effects therefore cannot be excluded.

Future studies should include adequately powered multicentre randomised trials or carefully designed prospective cohorts using severity matching, propensity-based adjustment, or multivariable modelling to reduce treatment-selection bias. Key endpoints should include mortality, survivor-conditional renal recovery, dialysis independence, long-term kidney function, adverse events, ICU and hospital length of stay, health-related quality of life, and resource utilisation. Biomarker-guided patient selection may help identify clinical phenotypes most likely to benefit from adsorptive hemofiltration. Economic evaluations should incorporate equipment, consumables, staffing, treatment duration, and total hospitalisation costs. Future investigations should also systematically assess the timing of both RRT and oXiris initiation, duration of adsorptive therapy, anticoagulation strategy, delivered treatment dose, modality selection across different levels of illness severity and healthcare-resource settings, and the influence of competing events and longitudinal missing data on clinical outcome estimates.

## Conclusions

This prospective observational study compared patients who received oXiris-based CRRT with those who received SLED. Patients in the oXiris-based CRRT group had a lower proportion surviving to hospital discharge without an ongoing requirement for RRT, higher ICU mortality, a longer ICU stay, and a longer duration of RRT, while patients receiving SLED showed more favourable unadjusted values for selected hemodynamic and metabolic measures at 72 hours. Because the renal endpoint required survival to hospital discharge, it should not be interpreted as renal recovery conditional on survival. The results should be interpreted with caution because patients selected for oXiris-based CRRT had greater baseline hemodynamic and organ-support requirements, including lower mean arterial pressure, substantially higher vasopressor requirements, and more frequent invasive mechanical ventilation. Although the baseline SOFA score was numerically higher in the oXiris-based CRRT group, the study was not powered to establish equivalence or comparability between groups on the basis of this non-significant difference. These outcome differences were unadjusted and cannot be attributed solely to RRT modality, particularly given the marked baseline imbalance and potential confounding by indication. Differences in ICU length of stay, RRT duration, and 72-hour measurements should also be interpreted cautiously because of differential mortality, available-case assessment, and unadjusted baseline differences. SLED may represent a feasible option for selected patients who can tolerate prolonged intermittent dialysis, particularly in settings where continuous therapy is less accessible. The present findings do not establish either the superiority of SLED or a lack of benefit from oXiris-based CRRT because the non-randomised design and baseline severity imbalance preclude causal inference. Treatment goals, organ dysfunction, hemodynamic condition, the need for continuous renal support, and available resources should be considered when selecting an RRT modality. Larger multicentre randomised trials or prospective severity-adjusted comparative studies are required to determine comparative effectiveness and identify patient subgroups most likely to benefit from adsorptive CRRT.
